# Adherence to Treatment Guidelines Among Patients With Type 2 Diabetes Attending a Tertiary Hospital in Jeddah, Saudi Arabia

**DOI:** 10.7759/cureus.73850

**Published:** 2024-11-17

**Authors:** Suhaib Radi, Hatem A Alsolami, Mahmoud W Bader, Mohammed K Almazmumi, Abdulrahman H Alsahafi, Jehad H Habeeballah, Rashed I Ibrahim, Hamad A Alosaimi, Jehad A Alzahrani, Mohamed E Ahmed

**Affiliations:** 1 Department of Endocrinology, Ministry of National Guard Health Affairs, Jeddah, SAU; 2 College of Medicine, King Saud Bin Abdulaziz University for Health Sciences, Jeddah, SAU; 3 College of Medicine, King Abdullah International Medical Research Center, Jeddah, SAU; 4 College of Sciences and Health Professions, King Saud Bin Abdulaziz University for Health Sciences, Jeddah, SAU

**Keywords:** ada guidelines, adherence to guidelines, american diabetic association, glucagon-like peptide 1 receptor agonist (glp-1a), sodium-glucose cotransporter 2 inhibitor (sglt2i), type 2 diabetes mellitus

## Abstract

Introduction: Type 2 diabetes mellitus (T2DM) is a chronic metabolic disease that is associated with many complications if untreated. Guidelines, including those from the American Diabetes Association (ADA), are published regularly to enhance the management of diabetes in all aspects of care including regular screening of diabetic complications and encouraging the use of newer medications such as sodium-glucose cotransporter 2 inhibitors (SGLT2i) and glucagon-like peptide 1 receptor agonists (GLP-1a). This study aims to assess the adherence to ADA guidelines at a tertiary care center in Saudi Arabia.

Methodology: The study employed an observational, retrospective chart review design, utilizing medical records and patient charts from the Diabetic Center at the National Guard Health Affairs (NGHA) Hospital in the western region. The study included a sample of 384 patients from a total population of 3,985 individuals. Participants were adults over 18 years of age with T2DM who were being treated at the diabetic center.

Results: The study included 384 patients, with 56% being female (215 patients), and the mean age was 62.8(±11) years. The mean duration of DM was 17.6(±9.6) years and the mean body mass index (BMI) was 31 kg/m^2^(±6.3). SGLT2i and GLP-1a were prescribed in 226 (59%) and 196 (51%) of cases, respectively. Two hundred and ninety-six (76.6%) patients measured their glycated hemoglobin (HbA1c) twice annually which meets with ADA recommendations, and the mean HbA1c was 7.8(±1.6) which is lower than two local and two regional studies. Blood pressure (BP) was controlled (<130/80) in 40% of patients, which is in between according to three local studies. Lipid profile was checked annually in 95% of patients, but only 27% met the ADA primary or secondary prevention goals. Regarding urine albumin-to-creatinine ratio (ACR), it was done annually in 75% of patients, and 89% of patients have seen an ophthalmologist which indicates better adherence than other studies.

Conclusion: The results conclude that the diabetic center at the NGHA Hospital in Jeddah generally provides good quality of care with high adherence to ADA guidelines; however, some aspects of care need to be improved including hypertension and dyslipidemia management. Moreover, further research should aim to be more specific when addressing the use of newer medications including SGLT2i and GLP-1a and its outcome in diabetes complications and patients' satisfaction.

## Introduction

Type 2 diabetes mellitus (T2DM) is a chronic metabolic disease with an insidious onset characterized by high blood glucose levels. Diabetes is one of the most prevalent diseases worldwide, especially in developed countries. The global incidence of T2DM has increased from 184.6 per 100,000 in 1990 to 259.9 per 100,000 in 2019 [1]. Moreover, in its 2022 report, the International Diabetes Federation (IDF) estimated that the global prevalence of diabetes among individuals aged 20-79 was 10.5% (representing 536.6 million people) in 2021, with projections indicating an increase to 12.2% (783.2 million people) by 2045 [2]. In addition, T2DM is a leading cause of morbidity and mortality, with patients typically developing cardiovascular disease (CVD) 14.6 years earlier than those without T2DM [3]. Diabetes is a leading cause of renal failure, causing 44% of all end-stage renal disease (ESRD) in the United States, and also the leading cause of lower limb amputation in the United States [3]. In 2017, T2DM was ranked the ninth leading cause of mortality worldwide with more than one million deaths [4]. Furthermore, diabetes is a costly disease not only on an individual level but also on the healthcare system. Medical expenditure was estimated to be 2.3 times higher for patients with diabetes. In 2017, the healthcare cost of diabetes in the United States was 237 billion dollars [5].

In addition to its clear impact on the global healthcare system, diabetes is also a significant public health issue in Saudi Arabia. Like many countries in the Arabian Peninsula, Saudi Arabia has witnessed a rapid rise in the prevalence of diabetes over the past few decades; Saudi Arabia ranks the second highest prevalence of diabetes in the Middle East and seventh in the world. In Saudi Arabia, around seven million people have diabetes, and three million are pre-diabetics [6]. It is estimated that the healthcare cost of diabetes in Saudi Arabia is likely to exceed 0.87 billion dollars [7].

There are various treatment options for T2DM, including lifestyle changes, oral and injectable medications, and insulin. In addition, evidence-based treatment guidelines are constantly being developed and updated. Several studies have demonstrated a significant decrease in macro- and microvascular complications in diabetic patients with controlled blood glucose levels [8-10]. Similarly, other studies have found significant improvement in long-term outcomes in patients achieving the three goals of treatment (i.e., glycated hemoglobin (HbA1c) <7%, low-density lipoprotein cholesterol (LDL-C) <100 mg/dl, and blood pressure (BP) <140/90 mmHg) [10-12]. Moreover, diabetic guidelines such as American Diabetes Association (ADA) guidelines are suggesting new diabetic treatments such as sodium-glucose cotransporter 2 inhibitors (SGLT2i) and glucagon-like peptide 1 receptor agonists (GLP-1a) as glucose-lowering agents for those with established CVD or multiple risk factors as both have been proven to decrease the risk of major cardiovascular events including myocardial infarction, stroke, and cardiovascular death [13]. Also, SGLT2i are suggested to be part of the management in heart failure patients as they have been proven to decrease cardiovascular mortality and symptoms in heart failure patients [13].

Despite available guidelines for T2DM management, several studies have reported suboptimal quality of care in both developed and developing countries [14-16]. However, there remains a paucity of data regarding the quality of care provided to diabetic patients in Saudi Arabia. Hence, the present study aims to assess adherence to treatment guidelines among patients with diabetes being treated at a tertiary university hospital in Jeddah, Saudi Arabia.

## Materials and methods

The study utilized an observational retrospective chart review design. The research was conducted at the Diabetic Center in the National Guard Health Affairs (NGHA) Hospital in Jeddah, which serves as a tertiary healthcare facility for patients with diabetes. The inclusion criteria involved adults aged 18 years and above who were diagnosed with T2DM and receiving care at the diabetic center. Patients who had been followed for less than one year were excluded from the study. A total of 384 participants were included in the study using convenience sampling. To prevent duplication, patients' medical record numbers (MRNs) were recorded on an electronic sheet with only access to the research team members. The sample size was determined using an equation based on a total population of 3,985 patients, resulting in a sample size of 384 patients.

The research received approval from the Institutional Review Board (IRB) of King Abdullah International Medical Research Center (KAIMRC) under the approval number IRB/2537/23. Data collection was conducted from November 4, 2023, to February 13, 2024. The electronic medical records from the BESTCare system served as the data source. An electronic questionnaire was developed based on previous studies [17], and it was reviewed by an expert endocrinologist. The questionnaire consisted of four parts. The first part covered demographic variables, including the patient's MRN, most responsible physician (MRP), age, gender, duration since diagnosis, weight, height, and body mass index (BMI). The second part focused on comorbidities such as hypertension, dyslipidemia, and heart failure, followed by the assessment of macrovascular and microvascular diabetic complications. The third part included the follow-up plan and outcomes, including the frequency of annual check-ups, the latest values of HbA1c, BP, serum creatinine, urine albumin-to-creatinine ratio (ACR), foot examinations, depression screening, and referrals to ophthalmology or nephrology. The fourth part encompassed the medications used by the patients, including diabetes medications, hypertension medications, dyslipidemia medications, and blood thinners.

JMP, Version 17 (SAS Institute Inc., Cary, North Carolina, United States), a statistical software, was used under the supervision of a biostatistician. In cases where calculating the ACR was difficult due to very high or very low albumin levels, an estimation was made. ACR was assumed to be 300 mg/mmol for patients with very high albumin levels and 0.5 mg/mmol for patients with very low albumin levels.

The collected data then were analyzed using various statistical tests to explore relationships, associations, and differences among the variables of interest. For descriptive statistics, the frequencies and percentages were used for qualitative data. The mean plus standard deviation and the median plus Q1-Q2 were used for quantitative data. For inferential statistics, to examine the association between two categorical variables consisting of two groups, the chi-squared test was used. A p-value of less than or equal to 0.05 is considered significant.

The collected data were stored in a secure file accessible only to the research team. Before analysis, the data were reviewed, and any identifying information such as MRNs and MRP details were deleted. After analysis, the data were deleted from the electronic sheet.

## Results

In this study, 384 patients were included, and the number of female patients was slightly higher with a total of 215 (56%) patients, and the mean age was 62.8(±11) years. The mean DM duration in years was 17.6(±9.6) years. Regarding BMI, the mean BMI was 31 kg/m^2^(±6.3). Also, in this study, multiple comorbidities have been assessed. The most prevalent comorbidity was dyslipidemia with a total of 323 patients (84%) followed by hypertension with 289 patients (75%). Regarding medications, the most prescribed anti-diabetic medication was metformin in 286 patients (74%), while SGLT2i and GLP-1a were prescribed in 226 (59%) and 196 (51%) of cases, respectively. More demographic details will be shown in Table 1.

**Table 1:** Demographic details BMI: body mass index; DDP-4i: dipeptidyl peptidase-4 inhibitors; SGLT2i: sodium-glucose cotransporter 2 inhibitors; GLP-1a: glucagon-like peptide 1 receptor agonists; ACEi: angiotensin-converting enzyme inhibitors; ARBs: angiotensin receptor blockers; CCBs: calcium channel blockers

Variable	Value n (%) or M±SD
Gender	
Male	169 (44%)
Female	215 (56%)
Age	62.8(±10.9)
Diabetes duration in years	17.6(±9.6)
Weight (kg)	80.1(±15.7)
Height (cm)	160.6(±9.6)
BMI	31.2(±6.3)
Diagnosed with	
Hypertension	289 (75%)
Dyslipidemia	323 (84%)
Heart failure	22 (5%)
Macrovascular complications	
Coronary artery disease	89 (23%)
Cerebrovascular disease	40 (10%)
Peripheral artery disease	20 (5%)
Not established macrovascular complication	260 (68%)
Microvascular complications	
Diabetic retinopathy	149 (39%)
Diabetic nephropathy	109 (28%)
Diabetic neuropathy	92 (24%)
Not established microvascular complication	156 (40%)
Anti-diabetic medications	
Metformin	286 (74%)
Sulfonylurea	79 (20%)
DDP-4i	66 (17%)
SGLT2i	226 (59%)
GLP-1a	196 (51%)
Insulin	264 (69%)
Anti-hypertensive medications	
ACEI/ARBs	250 (65%)
CCBs	104 (27%)
Diuretics	75 (20%)
Beta-blockers	66 (17%)
Lipid-lowering medications	
Statins	353 (92%)
Ezetimibe	100 (26%)
Evolocumab	16 (4%)
Antiplatelets	150 (39%)

Macrovascular complications in diabetes refer to the damage of large blood vessels, leading to coronary artery disease, cerebrovascular disease, and peripheral vascular disease (PVD) mainly to the lower limbs, while microvascular complications involve damage to small blood vessels, resulting in issues like diabetic retinopathy, diabetic nephropathy, and diabetic neuropathy. Regarding macrovascular complications, the most prevalent was coronary artery disease with a total of 89 (23%) patients followed by cerebrovascular disease and peripheral artery disease with 40 (10%) and 20 (5%) patients, respectively. Twenty-two patients (5%) had concomitant heart failure. Microvascular complications were more prevalent with diabetic retinopathy being the most common with a total of 149 (39%) patients followed by diabetic nephropathy and diabetic neuropathy with 109 (28%) and 92 (24%) patients, respectively. Two hundred and sixty (68%) patients did not have established macrovascular disease and 156 (40%) did not have established microvascular disease.

The next section is about adherence to ADA guidelines. ADA suggests that HbA1c should be measured at least twice annually and that represents 296 (76.6%) patients in this study and the mean HbA1c was 7.8(±1.6). Regarding lipid measurements, ADA suggests that the LDL target should be ≤1.7 mmol/L in primary prevention and ≤1.4 mmol/L in secondary prevention and this represents 103 (27%) patients in this study. Also, an LDL check is suggested to be done once annually, and it is done in 365 (95%) of patients. More details about ADA targets will be seen in Table 2 and the supplementary table found in the Appendices.

**Table 2:** Number of patients achieving the ADA guidelines HbA1c: glycated hemoglobin; LDL: low-density lipoprotein; ADA: American Diabetes Association

Aspect of care	Number of patients
HbA1c	
HbA1c ≤7	147 (38%)
Checked≥2 times annually	296 (76.6%)
Blood pressure	
Blood pressure ≤130/80	173 (40%)
LDL urine	
LDL ≤1.7 (primary prevention) or ≤1.4 (secondary prevention)	103 (27%)
Checked ≥1 annually	365 (95%)
Albumin-to-creatinine ratio	
Checked ≥1 annually	288 (75%)
Serum creatinine	
Checked ≥1 annually	381 (99%)
Foot examination	
Done ≥1 annually	66 (18%)
Depression screening	
Done ≥1 annually	52 (13%)
Ophthalmology visit/referral	
Done ≥1 annually	343 (89%)

**Figure 1:**
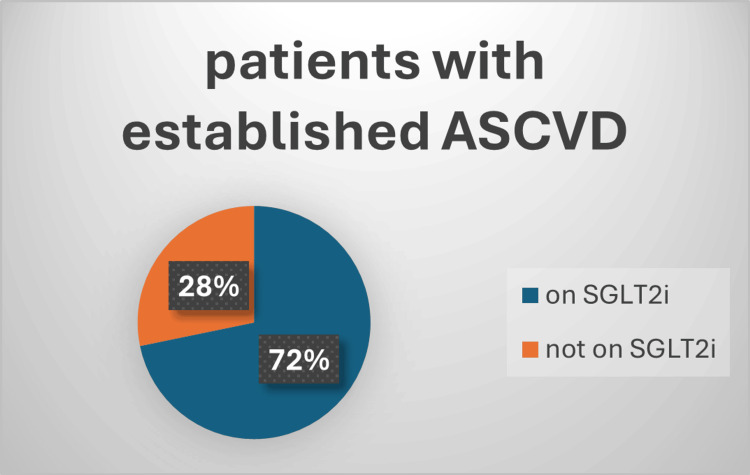
Percentage of patients on SGLT2i as an approved cardioprotective agent Chi square=12.6; p-value=0.0004 ASCVD: atherosclerotic cardiovascular disease; SGLT2i: sodium-glucose cotransporter 2 inhibitors

**Figure 2:**
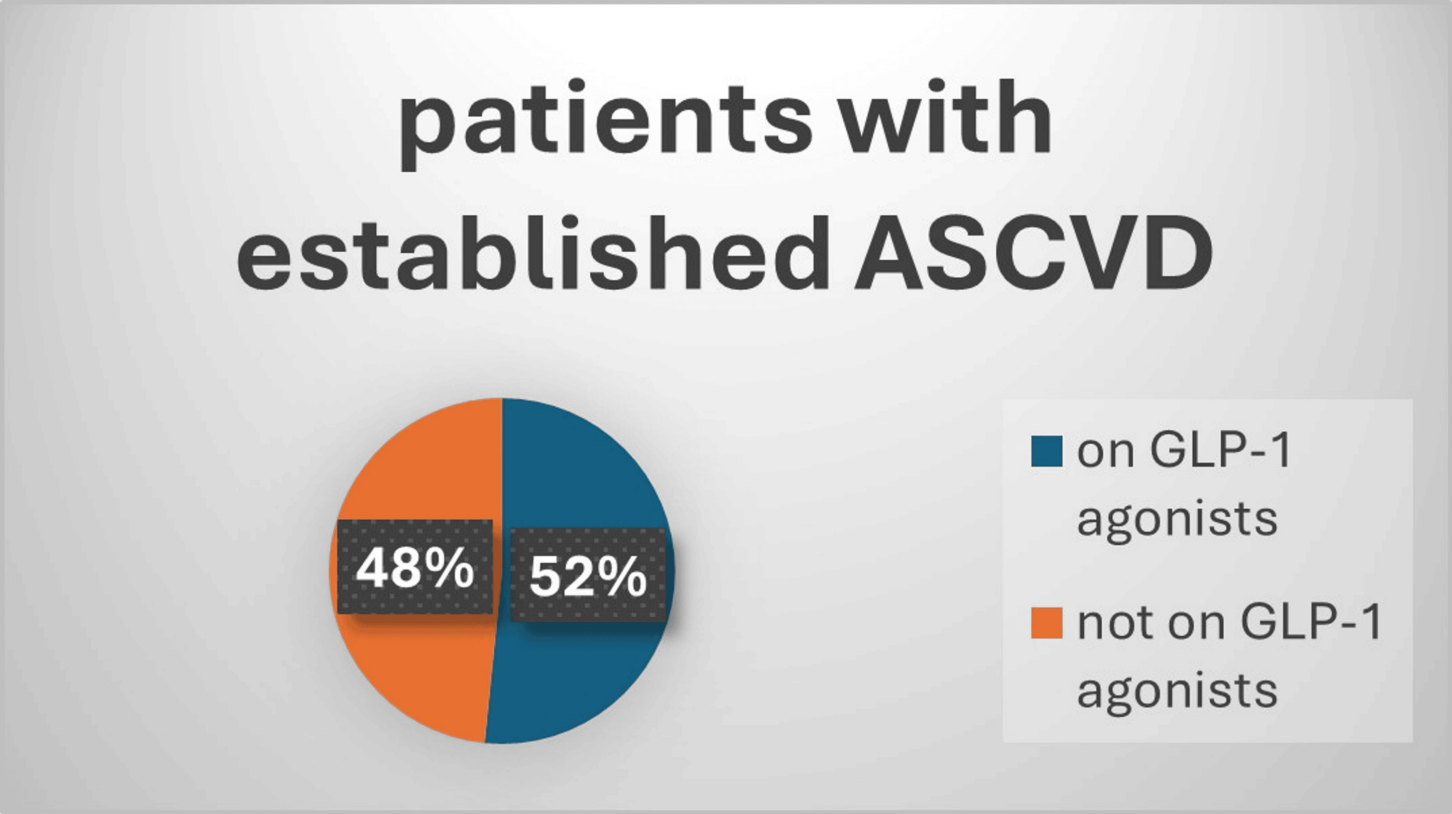
Percentage of patients on GLP-1a as an approved cardioprotective agent Chi square=0.45; p-value=0.5 ASCVD: atherosclerotic cardiovascular disease; GLP-1a: glucagon-like peptide 1 agonists

**Figure 3:**
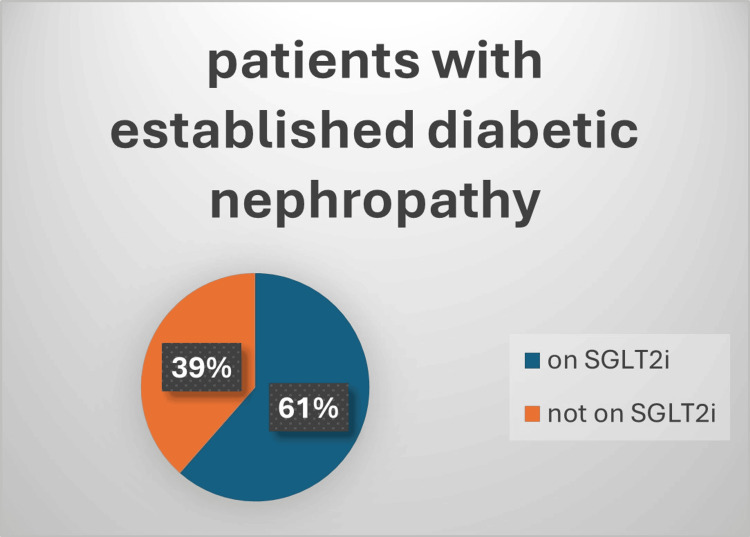
Percentage of patients on SGLT2i as an approved renoprotective agent Chi square=0.024; p-value=0.87 SGLT2i: sodium-glucose cotransporter 2 inhibitors

**Figure 4:**
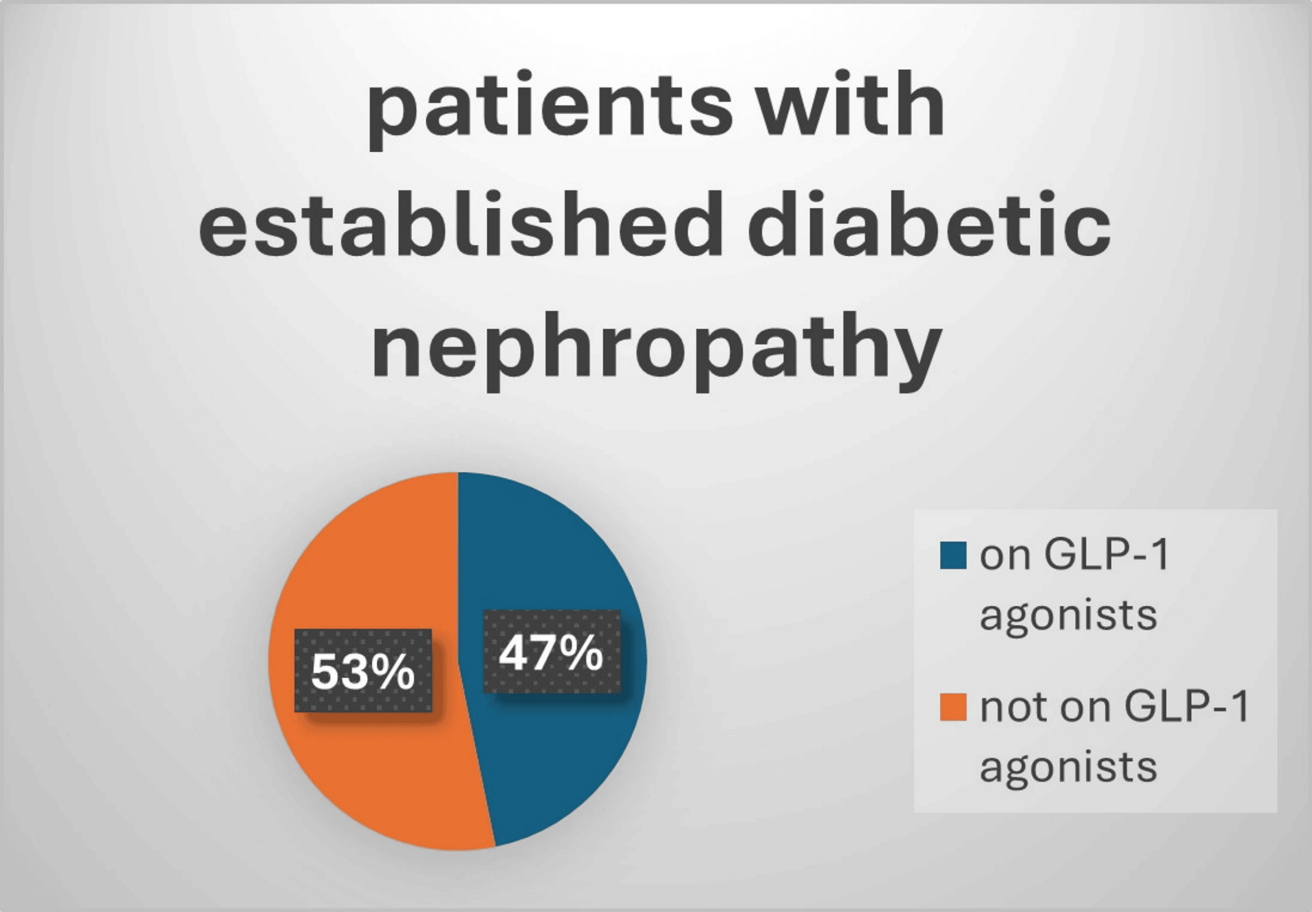
Percentage of patients on GLP-1a as an approved renoprotective agent Chi square=1.1; p-value=0.29 GLP-1a: glucagon-like peptide 1 agonists

One hundred and twenty-six patients had established atherosclerotic cardiovascular disease (ASCVD), 89 of them (72%) received SGLT2i and 64 (52%) of them received GLP-1a, and this is shown in Figure 1 and Figure 2. Also, 109 patients had documented diabetic retinopathy or chronic kidney disease (CKD), 61% of them received SGLT2i while 47% received GLP-1a, and that is shown in Figure 3 and Figure 4. Moreover, 22 patients were diagnosed with heart failure, and 16 (73%) of them were on SGLT2i.

## Discussion

In this study, we report the adherence to the ADA treatment guidelines among adults with T2DM in the diabetic center, where patients are being followed by the endocrinology department at the NGHA Hospital in Jeddah.

In terms of medical evaluation and assessment, the majority of our patients (76.6%) had their HbA1c level measured at least twice a year; this is greater than what was previously reported from another tertiary center (49.75%) [18]. The mean HbA1c of 7.8, in our study, is marginally lower compared to the results of studies conducted locally and regionally. Two local studies done in two different settings found a higher mean HbA1c: the first study done in primary healthcare centers (PHC) locally found the mean HbA1c to be in a range (8.09-8.32% in 2017 and 7.87-8.34% in 2018) [17], while another study done in a local hospital found the mean HbA1c to be 8.67% [18]. Regionally, the mean HbA1c was higher than our study in two studies conducted in the United Arab Emirates and Iran, at 8.3% and 8.01%, respectively [19,15]. Around 38% of patients in our clinics met the <7 HbA1c target; this is greater than the percentages observed in two local studies done under PHC settings (23.1-26.3% and 24.2%, respectively) [17,20]. Also, this is higher than the percentage found in another local hospital where 24.53% of patients achieved the target HbA1c. In a different local diabetic center, only 8.1% achieved an HbA1c of <7% [21]. While this is, as well, lower than our finding, 34% of patients in Iran achieved an HbA1C <7%; nevertheless, these data were based on a large number of participants under various clinic settings [15]. These results can be partially explained by the lower load and more specialized clinics in the endocrinology department in the diabetic center in comparison to PHCs, which have a higher load of patients, and also the availability of new medications including SGLT2i and GLP-1a especially when compared with old studies that were conducted when those medications were not available.

Regarding BP, it's a standard protocol to measure BP and other vitals with each visit. Our finding showed that 40% of our patients had controlled BP (<130/80), numbers that are in between and close to findings from PHCs [17,20]. However, it was higher when compared to another local hospital, having only 29.03% achieving controlled BP [18]. It should be noted that even with routine BP monitoring, less than half of our patients had controlled BP. Furthermore, just 39% of all patients were taking more than one medication, even though the majority of our patients still had uncontrolled BP. This could be attributed to the challenges that patients or physicians have in managing BP. Moreover, lower-risk patients can be considered to have controlled BP if it's under 140/90 so applying this threshold could also improve the number of patients with controlled BP. In addition, some patients might have white coat phenomena, making measuring the BP during the visit inaccurate, and in these cases, home monitoring is advisable. 

While 95% of our patients obtained an annual LDL check, only 27% of them met the ADA target for primary or secondary prevention (less than 1.7 mmol/L and less than 1.4 mmol/L, respectively). The annual monitoring rate in our center is greater than other local studies, which was 69.6% [20]. Additionally, our center's mean LDL level was lower compared to other regional studies (2.2 mmol/L vs 2.65 mmol/L in 2018) [17] and also lower in comparison to other endocrinology clinics at a local hospital with a mean of 2.9 mmol/L [18]. In comparison to other endocrinology clinics at local hospitals and a diabetic center, which had yearly checkup rates of 81.8% and 71.5%, respectively, both achieved higher LDL targets (51.1% and 48.8%, respectively); however, their results were based on higher targets (<2.6 mmol/L and <3.3 mmol/L, respectively) compared to our study [18,21]. It's worth mentioning that despite the low rate of patients achieving LDL targets, only around one-quarter of patients have additional treatment (ezetimibe and evolocumab) to their statin therapy which may suggest lower adherence in terms of lipid management.

For microvascular complication screening, ACR measurements for nephropathy screening are provided to 75% of our patients annually, and 89% of our patients saw an ophthalmologist for retinopathy. These results indicate better adherence than other local studies [18,20,21]. Also, regarding neuropathy and PVD, only 18% of patients had documented foot examinations, a comparable result to another diabetic center in the southern area [21]. However, it is significantly lower than the PHC where 72% of their patients had documented foot exams [20]. Similarly, depression screening is only done for 13% of patients. An important factor contributing to these relatively low adherence rates, especially to neuropathy and depression screening, is the lack of specialized clinics, particularly for foot care and psychological support, which limits our ability to provide comprehensive care and may lead to missed screenings. Additionally, time constraints may hinder thorough documentation and routine screening for complications like neuropathy and PVD. Implementing a standardized format for documentation and establishing dedicated clinics for foot care and psychological support could help improve adherence to screening guidelines and ensure a more holistic approach to diabetes management.

Regarding compliance with ADA guidelines for cardio-renoprotective treatment, around 32% of our patients have established CVD, and 28% have diabetic nephropathy. In terms of ASCVD, about half of ASCVD patients with an established disease are taking a GLP-1a (semaglutide), and 72% were taking SGLT2i (dapagliflozin). For heart failure (whether preserved or reduced), similarly around 73% were on SGLT2i. These findings were more favorable than those from the CAPTURE study, which included 13 different countries and found that just 21.9% of patients with CVD received medication with a cardiovascular benefit [22] and only 23.6% of patients in Saudi Arabia, which was also included in the CAPTURE study, were taking cardioprotective medications, with 11.6% using GLP-1a and 13.1% taking SGLT2i [23]. The CAPTURE study was based on data from 2019. Nevertheless, a more recent investigation revealed comparable findings, with around half of their ASCVD patients receiving cardioprotective antihyperglycemic medication [24]. It seems the discrepancy between the recent results, including ours, and the CAPTURE study was the limitation related to the availability of GLP-1a and SGLT2i in Saudi Arabia which is dependent upon purchasing decisions, policies, and prescribing privileges in individual governmental hospitals, as indicated in the CAPTURE study [22]. In assessing adherence to guidelines, a study conducted in the United Arab Emirates for T2DM patients with CVD showed that 56% of the patients received cardioprotective medications, of which 50% were on SGLT2i and 21.6% were on GLP-1a [19]. Remarkably, we can see that SGLT2i is used more frequently than GLP-1a in all of the previous results. We think there are a variety of factors that we observed in our clinics and most possibly adverse effects of GLP-1a, availability issues, or fear of beginning injectable medication by patients.

For renoprotective antihyperglycemic medication, out of 109 patients with diabetic nephropathy, 67 (61%) received SGLT2i, a result that is slightly lower than a recent study which found 73% of diabetic nephropathy patients receiving SGLT2i while obtaining care at PHC in Jazan City, Saudi Arabia [25]. The lower utilization of SGLT2i in our patients could be related to several factors, including limited awareness among healthcare providers, restrictions in prescribing practices, or patient-related factors.

These findings are consistent with recent evidence highlighting the importance of GLP-1a and SGLT2i as cardio-renoprotective treatment. For instance, the FLOW trial conducted by Perkovic et al. demonstrated that GLP-1a improve clinically significant kidney outcomes and reduce cardiovascular mortality in patients with T2DM and CKD [26]. Additionally, a recent meta-analysis by Apperloo et al. found that SGLT2i reduce the risk of cardiovascular events, heart failure hospitalization, and the progression of CKD [27].

Strengths, limitations, and recommendations

Our study was conducted in the endocrinology clinic at the NGHA Hospital with a sample size of 384 patients which showcases several strengths that enhance its credibility and contribution to diabetes care. Firstly, the comprehensive patient follow-up and data collection from various physicians' clinics ensured a robust evaluation of current management practices. This approach allows for a detailed examination of laboratory findings, medication adherence, and screening practices, providing valuable insights into the multifaceted nature of diabetes management within a tertiary care setting. Moreover, this study not only sheds light on the current practices but also establishes a foundation for future research to build upon, particularly in terms of exploring diverse methodologies and expanding to a broader demographic to enhance generalizability and impact. We also included the assessment of newer cardio-renoprotective agents like GLP-1a and SGLT2i.

Regarding the limitations, this cohort may not be representative of the general population in Saudi Arabia or globally, limiting the generalizability of our findings. Additionally, the study primarily focused on patient follow-up, current laboratory findings, medications, and screening practices for T2DM, which provides a broad overview of diabetes management but does not encompass all aspects of diabetes care. Moreover, to overcome bias in this study, some measures have been taken, such as the inclusion of all patients with T2DM who are attending the diabetic center clinics and have been following up for more than one year. Also, an equal distribution of data collection from different physicians' clinics took place in this study which may have contributed to lower rates of bias; however, due to the consecutive sampling technique, bias cannot be ignored in this study, and more studies could be done using random sampling technique.

Future research should aim to address these limitations by including larger and more diverse populations. More detailed investigations are needed to explore specific aspects of T2DM management, such as the utilization and outcomes and patients' satisfaction with using SGLT2i and GLP-1a. This will help to provide a more comprehensive understanding of diabetes care and its nuances in different patient populations.

## Conclusions

Our study highlights the adherence to the ADA treatment guidelines among adult patients with T2DM managed at a specialized endocrinology center. The results indicate that, in general, patients at our center receive a higher standard of care compared to other local and regional settings. The specialized nature of our clinic and the availability of newer medications, such as SGLT2i and GLP-1a, have contributed to better glycemic control and improved adherence to cardiovascular and renal protection guidelines.

Despite these favorable outcomes, there are still areas that require improvement. BP and lipid management, along with comprehensive screening for microvascular complications, need more focused efforts. The uptake of newer antihyperglycemic agents for both glycemic and weight management might improve further by addressing barriers such as medication availability, physician prescribing practices, and patient acceptance.

Overall, while our findings demonstrate positive trends in diabetes care, further studies are needed to explore and address the gaps in management, particularly in less specialized settings. This will ensure a more uniform and effective approach to diabetes care across different healthcare facilities.

## Appendices

**Table 3:** More detailed assessment of patients' care and outcome HbA1c: glycated hemoglobin; LDL: low-density lipoprotein; eGFR: estimated glomerular filtration rate

Variable	Last reading mean (±SD) or median (Q1-Q3)	Not done in the last year	Once	Twice	Thrice	More than three times
Number of visits in the last year	NA	6 (1%)	43 (11%)	215 (56%)	75 (19%)	45 (11%)
HbA1c	7.8(±1.6)	9 (2.3%)	79 (20%)	183 (47.6%)	84 (22%)	29 (7%)
Blood pressure	133/67(±18/14)	8 (2%)	37 (9.6%)	81 (21%)	69 (17%)	189 (49%)
LDL	2.2(±0.9)	19 (5%)	101 (26%)	162 (42%)	73 (19%)	28 (7%)
Urine albumin-to-creatinine ratio	2.4 (1.1-8.1)	96 (25%)	140 (36%)	99 (25%)	36 (9%)	12 (3%)
Serum creatinine	74 (61-91)	3 (0.1%)	57 (15%)	118 (30%)	78 (20%)	128 (33%)
eGFR	81(±24.6)					
Foot examination	NA	318 (82%)	50 (13%)	13 (2%)	1 (0.02%)	2 (0.05%)
Depression screening	NA	332 (86%)	52 (13%)			
Ophthalmology visit/referral	NA	41 (11%)	343 (89%)			
